# Toward Robust EEG Classification Using Adaptive CNN–Transformer and Inception Architectures and Signal Level Augmentation

**DOI:** 10.3390/s26144636

**Published:** 2026-07-22

**Authors:** Vikas Reddy Venkannagari, Shivansh Sharma, Parthan Olikkal, Dev Parikh, Jay Paun, Farshad Safavi, Ramana Vinjamuri

**Affiliations:** Department of Computer Science and Electrical Engineering, University of Maryland Baltimore County, Baltimore, MD 21250, USA; wh22215@umbc.edu (V.R.V.); shivans1@umbc.edu (S.S.); polikka1@umbc.edu (P.O.); dparikh3@umbc.edu (D.P.); mp11252@umbc.edu (J.P.); fsafavi1@umbc.edu (F.S.)

**Keywords:** electroencephalography, convolutional neural network, transformer, emotion recognition, P300-based deception detection, data augmentation

## Abstract

Non-invasive electroencephalography (EEG) enables practical brain-state monitoring for applications such as emotion recognition and event-related potential (ERP)-based deception detection. However, robust EEG classification remains challenging because of noise, non-stationarity, limited labeled data, and substantial inter-subject variability. In this work, we present a sensor-density-aware framework that applies different deep architectures to low- and high-channel EEG acquisition settings and augments the training data using a physiologically constrained signal-level procedure. For the 5-channel LieWaves dataset, the CNN–Transformer achieved 97.14±1.36% subject-dependent accuracy with augmentation, compared with 92.91±4.34% without augmentation. For the 62-channel SEED dataset, the Inception CNN achieved 98.52±0.79% with augmentation and 98.44±0.83% without augmentation. The improvement on LieWaves was statistically significant, whereas the small improvement on SEED was not statistically significant. Under subject-independent evaluation, performance was 57.83±8.96% on LieWaves with augmentation and 57.95±8.38% on SEED. These results demonstrate strong subject-dependent performance while confirming that cross-subject generalization remains challenging. Overall, the proposed framework combines sensor-density-aware architecture selection with signal-level augmentation and provides a systematic comparison of subject-dependent and subject-independent EEG classification.

## 1. Introduction

Electroencephalography (EEG) has become a central modality in brain–computer interface (BCI) research owing to its millisecond-level temporal resolution, non-invasive acquisition, and relatively low instrumentation cost [1,2]. Two tasks that have received sustained attention are EEG-based emotion recognition, which infers affective states from neural correlates of emotional processing, and deception detection, which identifies concealed information through event-related potential (ERP) components such as the P300 [2,3]. Both tasks have practical relevance in mental health monitoring, adaptive human–computer interaction, and forensic assessment, yet both remain far from solved.

The central difficulty is that EEG signals are strongly subject-specific. Individual differences in cortical anatomy, cognitive strategy, and scalp impedance produce distinct amplitude and frequency profiles even for the same stimulus, making it difficult to train a single model that generalizes across users [4,5]. This inter-subject variability is compounded by two practical constraints. First, EEG is collected under heterogeneous hardware conditions: consumer-grade wearables typically offer five or fewer channels, whereas clinical and research systems provide 32–128 channels. These regimes produce fundamentally different input structures, and a single architecture rarely handles both well. Second, labeled EEG data are expensive to acquire; subject-dependent training further concentrates this scarcity by restricting training data to a single individual.

Prior work has addressed these challenges in isolation. Hybrid CNN–Transformer models have shown strong subject-dependent performance on high-density data [6,7], and GAN-based augmentation has demonstrated modest improvements under limited data [4,8]. However, (i) architectures are rarely co-designed for the channel-density regime of the target dataset, (ii) GAN-based methods introduce generative artifacts that can corrupt physiologically discriminative signal structure, and (iii) systematic evaluation across both subject-dependent and subject-independent settings on multiple datasets is uncommon, leaving the practical operating range of proposed methods unclear.

To make the motivation and scope of the study explicit, Table 1 summarizes the motivation, novelty, aim, and measurable objectives of the proposed work.

We address these limitations with three coordinated contributions:

1. Two density-matched architectures. For low-channel EEG, we propose a lightweight CNN–Transformer (≈850 k parameters) in which a three-layer CNN encoder extracts short-range spatiotemporal features and a six-layer Transformer encoder models long-range dependencies via multi-head self-attention. For high-channel EEG, we propose an Inception-based CNN that processes the input through three parallel convolutional branches with kernel sizes {3,5,7}, enabling simultaneous capture of short- and long-range temporal structure without proportionally increasing depth.
2. A signal-level augmentation strategy. We expand each training trial fourfold by injecting high-frequency components (>45 Hz) extracted from randomly selected reference trials in the training set into a cleaned version of the source trial. For the EEG paradigms considered here, these components are treated as predominantly noise-like, while the procedure is designed to largely preserve the lower-frequency signal structure.
3. Systematic cross-paradigm evaluation. We evaluate both architectures using dataset-specific subject-dependent protocols and a subject-independent leave-one-subject-out protocol on the 5-channel LieWaves deception detection dataset [2] and the 62-channel SEED emotion recognition dataset [1]. This evaluation quantifies the performance gap between subject-dependent and subject-independent classification across the two EEG acquisition settings.

The CNN–Transformer achieved 97.14±1.36% subject-dependent accuracy on LieWaves, whereas the Inception CNN achieved 98.52±0.79% on SEED. On LieWaves, augmentation produced a statistically significant 4.23 percentage-point improvement in the subject-dependent setting. On SEED, the 0.08 percentage-point improvement was not statistically significant. Subject-independent performance decreased to 57.83±8.96% on LieWaves with augmentation and 57.95±8.38% on SEED, demonstrating that cross-subject generalization remains the primary limitation of the proposed framework.

## 2. Related Works

### 2.1. EEG-Based Emotion Recognition

Early EEG emotion recognition commonly relied on handcrafted spectral and spatial features, which were then classified using conventional machine learning models such as support vector machines (SVMs) and linear discriminant analysis (LDA) [5]. The introduction of deep learning shifted the field toward end-to-end representations. Zheng and Lu [1] established the SEED benchmark and demonstrated that differential entropy features across frequency bands, combined with deep belief networks, yield reliable three-class emotion discrimination. Subsequent work by Chakravarthi et al. [9] demonstrated that hybrid CNN–LSTM architectures achieve high classification accuracy by combining convolutional and recurrent feature learning for EEG-based emotion recognition. Zhang et al. [10] further proposed emotion aware embedding spaces to improve discrimination of underrepresented emotional categories.

Despite these advances, most prior studies adopt subject-independent evaluation without adequately characterizing the performance gap relative to subject-dependent baselines. As a result, the practical cost of cross-subject generalization is underreported, and architectural choices are rarely justified against the specific channel density of the target dataset.

### 2.2. EEG-Based Deception Detection

P300-based deception detection has a long history grounded in the concealed information test, where differential P300 amplitude to recognized versus unrecognized stimuli serves as a deception indicator [3]. Traditional approaches applied template-based matching and linear discriminant classifiers to trial-averaged ERP waveforms [11]. Aslan et al. [2] introduced the LieWaves benchmark and demonstrated that CNN–LSTM models operating on discrete wavelet transform features achieve up to 98.76% subject-dependent accuracy. However, prior work on LieWaves operates exclusively in a subject-dependent setting and relies on explicit feature engineering rather than end-to-end learning from raw signals.

### 2.3. Data Augmentation for EEG

Data scarcity is a persistent bottleneck in EEG-based deep learning because signal collection is time consuming and subject-dependent training further restricts available samples per model. GAN-based augmentation has been explored extensively: Zhang et al. [4] applied a conditional Wasserstein GAN to synthesize emotion-labeled EEG trials, and Bao et al. [8] used similar generative strategies to improve three-class classification on SEED. While effective in aggregate, generative augmentation carries inherent risks; synthesized signals may drift from the physiological manifold, introduce label inconsistencies, or corrupt discriminative low-frequency structure.

An alternative is signal-level augmentation that operates directly on recorded trials. The EEG-Inception paper [12] proposed injecting noise derived from reference EEG segments into cleaned trials, reporting a 3–5% accuracy improvement. We adopt and extend this strategy by restricting noise extraction to components above 45 Hz via an 8th-order Butterworth high-pass filter, ensuring that augmentation modifies only high-frequency texture which carries no physiologically significant EEG information while leaving class-discriminative spectral content intact.

### 2.4. Hybrid Deep Learning Architectures for EEG

CNNs are effective at capturing local spatiotemporal structure in multi-channel EEG due to their weight sharing inductive bias across the time and channel axes [12]. Transformers, originally developed for sequence modeling in natural language processing [13], address the complementary limitation of CNNs by modeling long-range dependencies through scaled dot-product self-attention. Devarajan et al. [6] demonstrated that combining convolutional feature extraction with Transformer encoding yields consistent gains over either component alone on SEED-style benchmarks. Li et al. [7] proposed a CNN-Informer variant for EEG seizure detection, achieving efficient long-range dependency modeling at reduced computational cost. Another approach utilizing a transformer-based architecture [14], designed to fuse EEG and EMG signals for motor control in rehabilitation tasks, achieved an accuracy of 87.27% across multiple levels.

**Figure 1:**
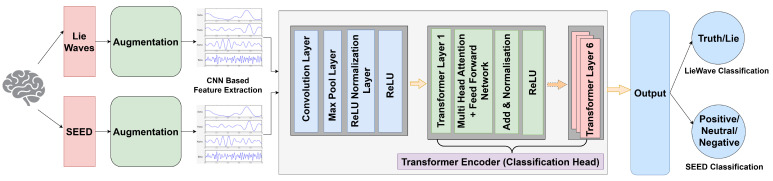
End-to-End Architecture of a hybrid CNN–Transformer, detailing the augmentation of lie waves and SEED EEG data, CNN-based feature extraction, and subsequent multi-task classification.

For high-density multi-channel data, Inception-style architectures [15] offer an appealing alternative to depth-stacking: parallel branches with heterogeneous kernel sizes capture features at multiple temporal scales simultaneously, widening representational capacity without proportionally increasing parameter count. This property is particularly well matched to EEG, whose discriminative structure spans a broad frequency range (delta through gamma bands). Prior applications of Inception-based designs to 1D biomedical signals [12] support this hypothesis, though no prior work has systematically compared CNN–Transformer and Inception architectures across the low and high density EEG regimes that characterize real-world deployment. The overall sensor-density-aware EEG classification framework is illustrated in Figure 1.

### 2.5. Subject-Dependent vs. Subject-Independent Evaluation

A growing body of work has documented the substantial performance gap between subject-dependent and subject-independent EEG models [1,2]. This gap reflects the well-established neurophysiological fact that intra-subject EEG variability is significantly lower than inter-subject variability [16], a property so pronounced that P300 ERPs have been used for biometric identification [17]. Domain adaptation [18] and multi-source transfer learning [19] are the most promising strategies for closing this gap, with reported improvements of up to 12.7% over non-transfer baselines on SEED. However, these methods introduce additional complexity and typically require either labeled target data or domain-adversarial training. In this work, we report both subject-dependent and subject-independent results to provide a complete performance profile, while focusing our methodological contributions on improving subject-dependent accuracy and robustness under limited data.

### 2.6. Critical Summary of Gaps in Existing Literature

Table 2 summarizes the main limitations observed in prior EEG classification literature and how the present work addresses them. This summary motivates the proposed sensor-density-aware architecture selection, signal-level augmentation, and dual subject-dependent/subject-independent evaluation design.

## 3. Problem Formulation

Let $X^{\left(i\right)}\in R^{C\times T}$ denote the raw EEG recording for trial *i*, where *C* is the number of channels and *T* is the number of time samples. Each trial carries a ground-truth label $y^{\left(i\right)}$ drawn from a task-specific label set: $y^{\left(i\right)}\in \left\{0,1\right\}$ for binary deception detection (LieWaves) and $y^{\left(i\right)}\in \left\{0,1,2\right\}$ for three-class emotion recognition (SEED).

Each recording is segmented into fixed-length epochs using a sliding window of length *L* and stride *S*, yielding an ordered set of epochs:

(1) $$E^{\left(i\right)}=\left\{x_{t}^{\left(i\right)}=X^{\left(i\right)}\left[:,\;tS:tS+L\right]\;|\;t=0,1,\ldots ,n_{i}-1\right\}$$

where $n_{i}$ is the number of valid windows for trial *i*. All epochs from a trial inherit the trial-level label $y^{\left(i\right)}$.

Given a training set $D_{\mathrm{train}}=\left\{\left(x_{t}^{\left(i\right)},\;y^{\left(i\right)}\right)\right\}$, the objective is to learn a parameterized classifier $f_{\theta }:R^{C\times L}\to Y$ that minimizes the empirical risk:

(2) $$\theta^{*}=\arg\min_{\theta }\;\frac{1}{|D_{\mathrm{train}}|}\sum_{\left(x,y\right)\in D_{\mathrm{train}}}L\;\left(f_{\theta }\left(x\right),\;y\right)$$

where L is the binary cross-entropy loss for LieWaves and the categorical cross-entropy loss for SEED. At inference, the predicted label for a new epoch x is:

(3) $$\hat{y}=\arg\max_{k\in Y}\;{\left[f_{\theta }\left(x\right)\right]}_{k}$$

**Figure 2:**
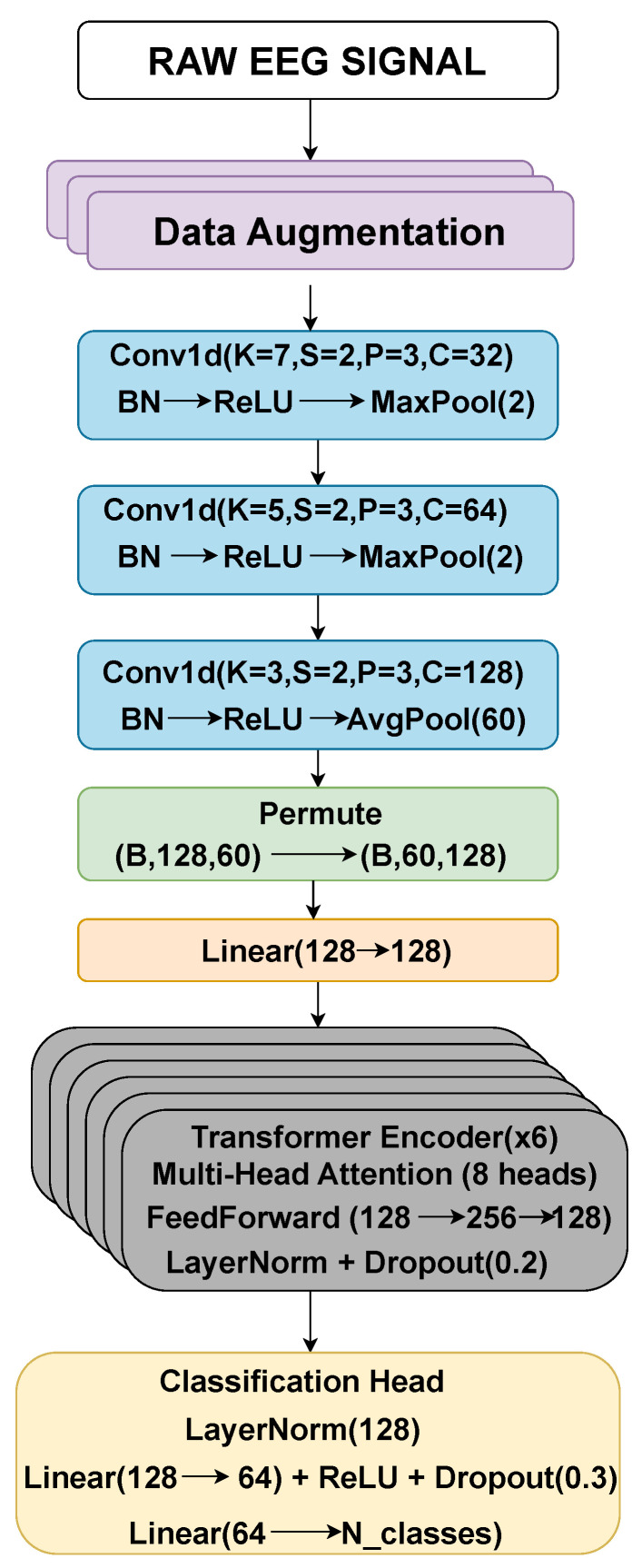
The end-to-end architecture of a hybrid deep learning model, detailing the 1D–CNN feature extractor and Transformer encoder for LieWaves EEG signal analysis.

The two evaluation paradigms differ in how the training and evaluation samples are selected. In the subject-dependent setting, separate models are trained for individual subjects using windows from the same subject. In the subject-independent setting, a leave-one-subject-out (LOSO) protocol is used: the model is trained on all subjects except one and evaluated on the completely held-out subject. The precise window-generation and fold-assignment procedures are described in Section 5. The detailed CNN–Transformer architecture used for LieWaves classification is shown in Figure 2.

## 4. Proposed Architectures

The two architectures share a common design philosophy: convolutional layers perform local temporal and channel-wise feature extraction, whereas the subsequent Transformer or Inception modules aggregate information over broader temporal contexts. The architecture is selected according to the channel-density regime of the dataset. For the low-density 5-channel LieWaves setting, a compact CNN–Transformer emphasizes temporal dependency modeling. For the high-density 62-channel SEED setting, an Inception CNN uses parallel temporal kernels to process the richer multichannel input. Therefore, the term sensor-density-aware refers to an explicit architecture-selection strategy rather than to an architecture that dynamically changes its structure during training.

### 4.1. CNN–Transformer for Low-Channel EEG

Figure 1 illustrates the full CNN–Transformer pipeline. The model processes input epochs of shape (B,C,L)=(B,5,128) and produces a scalar logit for binary classification. The layer-wise specification and parameter counts of the CNN–Transformer are summarized in Table 3.

#### 4.1.1. CNN Encoder

The CNN encoder extracts short-range spatiotemporal features through three sequential 1D convolutional layers. Filter counts increase progressively (32→64→128) while kernel sizes decrease (7→5→3), enabling early layers to capture coarse temporal structure and later layers to refine local discriminative patterns. Each convolutional layer is followed by batch normalization, ReLU activation, and max pooling. Batch normalization stabilizes intermediate feature distributions during mini-batch training and can improve optimization convergence. An adaptive average pooling layer after the third convolution standardizes the temporal dimension to 60, yielding an encoder output of shape (B,128,60).

#### 4.1.2. Transformer Encoder

The Transformer encoder receives the CNN output reshaped to (B,60,128), treating the 60 temporal positions as sequence tokens and the 128 feature maps as token embeddings. A linear projection layer re-scales token embeddings before self-attention. Positional embeddings were evaluated but did not improve performance, consistent with observations in prior EEG Transformer work [7], and were therefore omitted.

The encoder comprises six stacked layers, each containing: (i) multi-head self-attention with 8 heads of dimension 16, enabling the model to attend jointly to multiple temporal positions; and (ii) a position-wise feed-forward network (128→256→128) with ReLU activation. Residual connections and layer normalization are applied around both sub-layers. Dropout of 0.2 is applied to attention weights and feed-forward outputs. The six-layer encoder configuration was selected empirically during model development to balance representational capacity and parameter count.

#### 4.1.3. Classification Head

The Transformer output of shape (B,60,128) is mean-pooled along the sequence dimension to yield a (B,128) representation. Two linear layers (128→64→1) with ReLU activation, layer normalization, and dropout of 0.3 produce the final logit. A sigmoid activation maps the logit to a probability, with a threshold of 0.5 used for binary prediction.

### 4.2. Inception-Based CNN for High-Channel EEG

The Inception model processes input epochs of shape (B,62,400) and produces a three-class logit vector for emotion classification. The architecture adapts the Inception design principle [15] to 1D temporal EEG data, drawing on the EEG-Inception formulation of [12].

#### 4.2.1. Inception Block

Each Inception block applies three parallel 1D convolutional branches with kernel sizes 3, 5, and 7 to the same input. All branches use the same number of filters *F*, and their outputs are concatenated along the channel axis, yielding 3F feature maps. This design allows the model to capture local temporal patterns at multiple short time scales without committing to a single convolutional kernel size. Each Inception block is followed by batch normalization and ReLU activation.

#### 4.2.2. Full Architecture

Three Inception blocks are stacked with increasing filter counts (F=32,64,128), producing concatenated channel dimensions of 96, 192, and 384 respectively. Max pooling layers with kernel size 2 are applied after the first and second Inception blocks, reducing the temporal dimension from 400 to 200 and then to 100. The complete stage-wise shape transformation is given in Table 4. A global average pooling layer collapses the temporal dimension of the final (B,384,100) feature map to (B,384), which is passed to a fully connected layer producing three-class logits.

#### 4.2.3. Design Rationale

The Inception architecture is preferred over the CNN–Transformer for high-channel data for two reasons. First, with 62 input channels the spatial dimension already provides rich representational capacity, the primary modeling challenge is multi-scale temporal integration, which parallel Inception branches address directly without the quadratic attention cost of a Transformer. Second, the Inception design widens the network laterally rather than deepening it, reducing the risk of overfitting on a dataset with 15 subjects. The resulting model has 0.49 M parameters, remaining computationally lightweight despite the higher input dimensionality. The three-block configuration was selected empirically to progressively increase representational capacity while retaining a compact parameter count. The layer-wise characteristics and trainable parameter counts of the SEED Inception CNN are summarized in Table 5.

The computational profile of the Inception CNN is reported in Table 6.

The profiling results show that the Inception CNN contains fewer than 0.5 million trainable parameters, requires approximately 67.70 million multiply–accumulate operations per input window, and performs batch-size-one inference in less than 1 ms on the evaluated GPU.

## 5. Experimental Setup

### 5.1. Datasets

Table 7 summarizes the source, acquisition properties, task labels, and evaluation usage of the two datasets. Both datasets are de-identified EEG datasets used for academic research; therefore, no personally identifiable information is used in the present experiments.

#### 5.1.1. LieWaves

The LieWaves dataset [2] was collected using an Emotiv EEG headset recording from five channels (AF3, T7, Pz, T8, AF4) at 128 Hz following the international 10–20 placement system. Twenty-seven participants completed two counterbalanced sessions: a truth-telling session and a deception session. In each session, participants selected two beads from a set of ten (five designated per condition) and responded to visual stimuli by pressing a binary response button. The stimulus protocol consisted of 25 trials, each comprising a 3 s fixation screen, a 2 s bead image, and a 1 s inter-stimulus interval; the initial fixation period was excluded from analysis, yielding 75 s of stimulus-locked EEG per session. The final dataset has dimensions 27×2×5×9600 (subjects × sessions × channels × samples at 128 Hz).

#### 5.1.2. SEED

The SEED dataset [1] is a three-class emotion recognition benchmark comprising EEG recordings from 15 subjects across 15 trials each. Emotional states (positive, negative, neutral) were elicited via film clips, with no two consecutive trials sharing the same emotional category. Each trial consisted of a 5 s preparation cue, a 4-min film clip, a 45 s self-assessment period, and a 15 s rest interval. EEG was recorded from 62 channels at 1000 Hz; we use the provider-released recordings downsampled to 200 Hz. In the evaluated pipeline, each continuous trial recording was divided into 2 s windows of 400 samples using a stride of 200 samples, corresponding to 50% temporal overlap. The resulting input epochs have shape (62,400).

### 5.2. Preprocessing

#### 5.2.1. Segmentation

LieWaves recordings are segmented into 1 s epochs (L=128 samples) with a stride of S=96 samples (25% overlap), yielding approximately $n_{i}$ epochs per trial. SEED recordings use the provider-supplied 2 s segmentation (L=400 samples) with a stride of S≈200 samples (50% overlap). This yields input tensors of shape (5,128) for LieWaves and (62,400) for SEED.

#### 5.2.2. Normalization

Each epoch is normalized channel-wise to zero mean and unit variance using statistics computed exclusively from the training set:

(4) $$\begin{array}{cc} {\tilde{x}}_{c} & =\frac{x_{c}-\mu_{c}}{\sigma_{c}},\;\mu_{c}=\frac{1}{|D_{\mathrm{train}}|}\sum_{\left(x,y\right)\in D_{\mathrm{train}}}x_{c},\\ \sigma_{c} & ={\mathrm{Std}}_{\left(x,y\right)\in D_{\mathrm{train}}}\;\left(x_{c}\right) \end{array}$$

Validation and test epochs are transformed using the stored training statistics $\left(\mu_{c},\sigma_{c}\right)$, preventing any leakage of validation distribution information into the normalization step. Channel-wise normalization mitigates inter-subject amplitude variation and accelerates gradient convergence by placing all channels on a common scale.

### 5.3. Data Augmentation

To expand the training set without introducing generative artifacts, we propose a signal-level augmentation strategy based on high-frequency noise injection. The procedure operates as follows.

For each training trial $X^{\left(i\right)}\in R^{C\times T}$, three reference trials ${\left\{Y_{k}^{\left(i\right)}\right\}}_{k=1}^{3}$ are selected uniformly at random from the training set. A noise extraction operator Δ(·) is defined as an 8th-order Butterworth high-pass filter with cutoff frequency isolating components above 45 Hz, which are commonly treated as predominantly noise-like in scalp EEG for the paradigms considered in this study. The noise components of the source and reference trials are extracted as:

(5) $$N_{x}^{\left(i\right)}=\Delta \;\left(X^{\left(i\right)}\right),\;N_{y,k}^{\left(i\right)}=\Delta \;\left(Y_{k}^{\left(i\right)}\right),\;k=1,2,3$$

The cleaned source trial is obtained by subtracting its own high-frequency component:

(6) $$X_{\mathrm{clean}}^{\left(i\right)}=X^{\left(i\right)}-N_{x}^{\left(i\right)}$$

Each augmented trial is formed by adding one reference noise component to the cleaned source:

(7) $${\tilde{X}}_{k}^{\left(i\right)}=X_{\mathrm{clean}}^{\left(i\right)}+N_{y,k}^{\left(i\right)},\;k=1,2,3$$

This yields three augmented trials per original trial, quadrupling the effective training set size. All augmented trials inherit the label $y^{\left(i\right)}$ of the source trial. Because the perturbation is restricted to components above 45 Hz, the class-discriminative low-frequency structure of the signal is preserved exactly.

We evaluated two variants: (i) unrestricted reference selection, where $Y_{k}^{\left(i\right)}$ may be drawn from any class; and (ii) class-restricted selection, where references are drawn only from trials sharing the label $y^{\left(i\right)}$. No performance difference was observed between the two variants, suggesting that, for these datasets and preprocessing settings, components above 45 Hz contribute minimally to class discrimination. The unrestricted variant is used in all reported experiments. Augmentation is applied exclusively to training epochs; validation and test sets are never augmented.

The dataset sizes before and after augmentation are summarized in Table 8.

### 5.4. Training Protocol

All models were trained using the Adam optimizer with weight decay λ=0.01. Learning rates were tuned independently per architecture: $\eta =1\times 10^{-4}$ for the CNN–Transformer and $\eta =5\times 10^{-4}$ for the Inception model, selected from the candidate set $\left\{10^{-4},5\times 10^{-4},10^{-3},5\times 10^{-3}\right\}$ based on validation loss. A batch size of 32 was used throughout.

Training was regularized via early stopping with a patience of 10 epochs; the top three checkpoints ranked by validation accuracy were saved, and the best checkpoint was used for final evaluation. Dropout rates were set to 0.2 in the Transformer encoder and 0.3 in the classification head, determined empirically to minimize the gap between training and validation accuracy.

For the LieWaves binary task, training minimized binary cross-entropy loss with a sigmoid output activation; predictions were thresholded at 0.5. For the SEED three-class task, categorical cross-entropy loss was used with a softmax output activation. The evaluated search space and final selected hyperparameters are summarized in Table 9.

Learning rate had the strongest effect on convergence stability. Larger values accelerated early training but produced unstable validation loss, particularly for the CNN–Transformer. Dropout mainly controlled the train–validation gap, while the number of augmented copies controlled the trade-off between additional training diversity and diminishing returns. The final hyperparameters were selected based on validation accuracy, validation loss stability, and model compactness.

### 5.5. Evaluation Protocol

1. Assign each subject/trial to the appropriate training, validation, or held-out test partition according to the subject-dependent or LOSO protocol.
2. Generate sliding windows only within each already-assigned partition.
3. Fit normalization statistics using the training partition only.
4. Apply the stored training normalization statistics to validation and test windows.
5. Apply signal-level augmentation only to the training windows/trials.
6. Evaluate the final model only on unaugmented validation or held-out test data.

Table 10 summarizes the training, validation, and testing strategies used for the two evaluation settings. All splitting was performed before augmentation so that augmented samples could not leak into validation or test sets. To avoid leakage from overlapping EEG windows, all train–validation and train–test partitions were created at the original trial/subject level before window generation and before augmentation. After a trial was assigned to a split, all windows derived from that trial remained within the same split. Therefore, overlapping windows from the same original EEG trial could not appear simultaneously in training and validation/test sets. No random window-level split was used. Augmentation was applied only after this split and only to the training partition.

Subject-dependent evaluation. Separate models were trained and evaluated for each subject. For LieWaves, subject-specific data were divided into training and evaluation partitions using the dataset-specific split described above. For SEED, subject-level performance was estimated using ten-fold cross-validation. Per-subject accuracy, precision, recall, and F1-score were computed, and the mean and standard deviation across subjects were reported.

Subject-independent evaluation. A leave-one-subject-out (LOSO) protocol is applied: the model is trained on data from all subjects except one and evaluated on the held-out subject. This is repeated for each subject, and mean accuracy across folds is reported.

Statistical analysis. Paired subject-level accuracies obtained with and without augmentation were compared using a two-sided Wilcoxon signed-rank test [20]. Differences in inter-subject dispersion were evaluated using the Brown–Forsythe test [21], implemented as a median-centered Levene test. All tests were performed using SciPy, with the significance threshold set to α=0.05. Exact test statistics and *p*-values are reported in Section 6.

**Figure 3:**
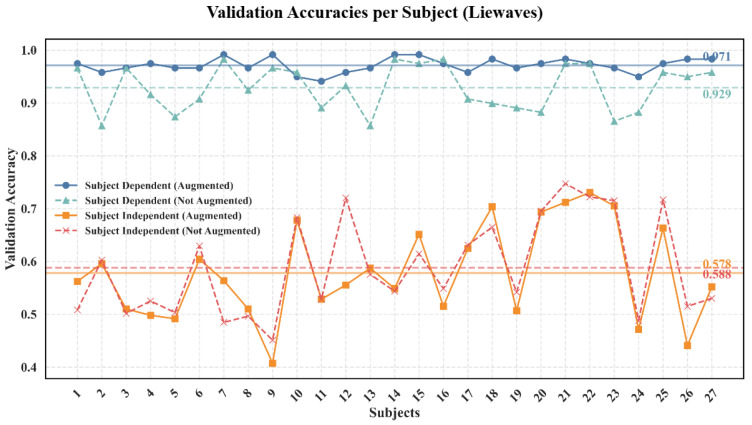
LieWaves accuracy comparison, illustrates the performance of a system under different conditions for 27 subjects (s1 to s27).

The final architectural configurations were selected empirically during model development based on validation behavior and model compactness. Complete controlled depth comparisons were not retained for all candidate configurations. Accordingly, systematic Transformer-depth and Inception-block-depth analyses are identified as future work. The subject-wise LieWaves accuracies under the four evaluated conditions are shown in Figure 3.

Figure 3 compares subject-wise LieWaves accuracy under subject-dependent and subject-independent evaluation, with and without augmentation. Solid curves represent results obtained with augmentation, whereas dashed curves represent results without augmentation. The upper curves correspond to subject-dependent evaluation, while the lower curves correspond to subject-independent evaluation. The horizontal lines indicate the mean accuracy across all 27 subjects for each condition.

**Figure 4:**
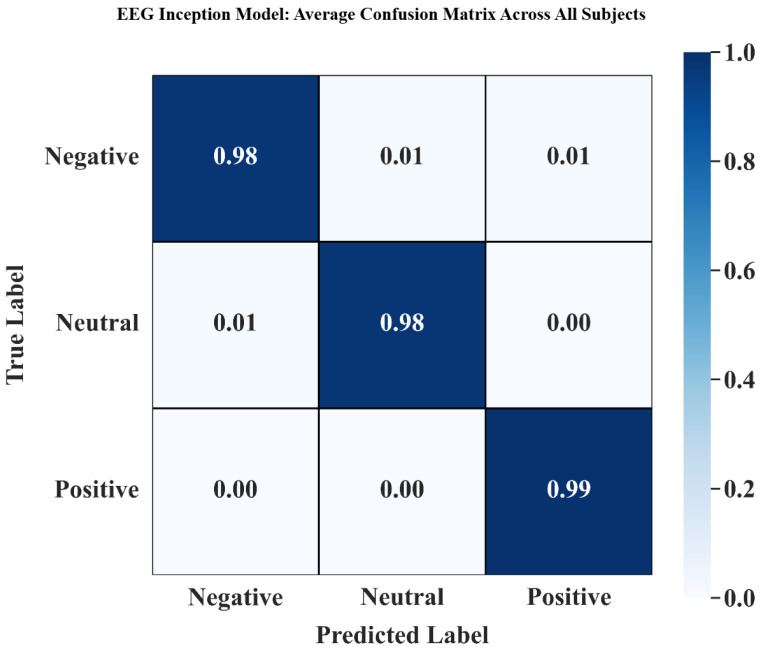
Inception model’s absolute-count confusion matrix on the SEED dataset under subject-dependent evaluation.

The subject-dependent confusion matrix of the SEED Inception CNN is shown in Figure 4.

### 5.6. Implementation Details

All experiments and inference profiling were conducted on a workstation equipped with an NVIDIA GeForce RTX 4070 Ti SUPER GPU with 16 GB of GDDR6X memory. Models were implemented in Python version 3.12.0 using PyTorch version 2.7.0 with CUDA 12.6; preprocessing and statistical analysis used NumPy version 2.1.3 and SciPy version 1.15.2. The two models contain approximately 0.49 M and 0.86 M trainable parameters, respectively, and training completed within a few hours per dataset on the described hardware. Code and experiment scripts are available upon request.

## 6. Results

The results are organized according to the objectives stated in Table 1: subject-dependent classification performance (O4), augmentation impact (O3), LOSO generalization (O5), and architecture and computational analysis (O1, O2, and O6). Unless stated otherwise, reported values are mean scores across subjects or folds.

### 6.1. Subject-Dependent Performance

#### 6.1.1. SEED Emotion Recognition

Table 11 reports subject-dependent accuracy, standard deviation, and F1-score for our Inception model against four prior architectures evaluated on SEED. Our model achieves 98.52% mean accuracy with a standard deviation of 0.79. Notably, the 3D-CNN + ELM model [22], despite achieving 90.85% accuracy, exhibits a standard deviation of 14.72 nearly twenty times larger than ours indicating that high mean accuracy and consistent cross-subject performance are not jointly guaranteed by prior architectures. The Deep Capsule Network [23] achieves the closest mean accuracy at 98.21% but does not report variance, making a robustness comparison impossible. Because several prior studies did not report subject-level variance, comparisons of inter-subject consistency remain incomplete.

Effect of augmentation on SEED. The mean subject-dependent accuracy changed slightly from 98.44±0.83% without augmentation to 98.52±0.79% with augmentation. A two-sided Wilcoxon signed-rank test showed that this difference was not statistically significant (W=36.0, p=0.1876). Similarly, the Brown–Forsythe test showed no significant difference in inter-subject dispersion (F(1,28)=0.1254, p=0.7259). These results suggest that augmentation had a limited effect on SEED, likely because the baseline accuracy was already near ceiling.

**Figure 5:**
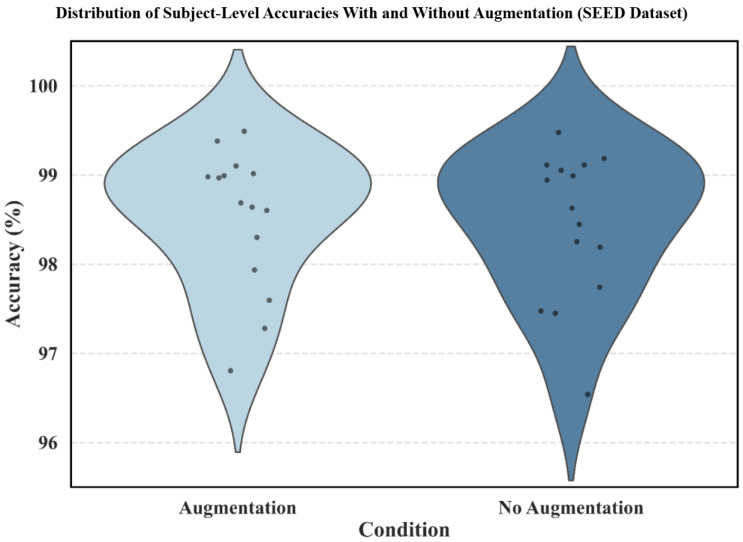
Distribution of subject-level SEED accuracies with and without augmentation. The narrower distribution under augmentation indicates reduced inter-subject variability in the subject-dependent setting.

The subject-level SEED accuracy distributions with and without augmentation are shown in Figure 5.

#### 6.1.2. LieWaves Deception Detection

Table 12 reports subject-dependent performance on LieWaves. The strongest prior result is a CNN–LSTM model [2] that uses Discrete Wavelet Transform (DWT) features as input, achieving 98.76% accuracy. Our CNN–Transformer, operating directly on raw EEG without manual feature engineering, achieves 97.14% accuracy and an F1-score of 0.97, closing most of the gap to the DWT-based method while eliminating the feature extraction pipeline entirely.

Effect of augmentation on LieWaves. Under subject-dependent evaluation, augmentation increased mean accuracy from 92.91±4.34% to 97.14±1.36%, corresponding to an absolute improvement of 4.23 percentage points. Accuracy improved for 23 of the 27 subjects, declined for two subjects, and remained unchanged for two subjects. A two-sided Wilcoxon signed-rank test showed that the paired improvement was statistically significant (W=6.0, $p=2.51\times 10^{-5}$). The Brown–Forsythe test also showed a statistically significant reduction in inter-subject dispersion, F(1,52)=48.792, $p=5.23\times 10^{-9}$.

Under subject-independent evaluation, augmentation changed the mean LieWaves accuracy from 58.85±9.10% to 57.83±8.96%. This difference was not statistically significant (W=135.5, p=0.3096), and the Brown–Forsythe test did not identify a significant difference in dispersion (F(1,52)=0.0879, p=0.7680).

### 6.2. Subject-Independent Performance

Under LOSO evaluation, the CNN–Transformer achieved 58.85±9.10% on LieWaves without augmentation and 57.83±8.96% with augmentation. The Inception CNN achieved 57.95±8.38% on SEED with augmentation. Table 13 provides a contextual comparison with previous subject-independent studies.

The studies listed in Table 13 differ in dataset, number of classes, input representation, use of domain adaptation, and evaluation protocol. Therefore, the table is intended to provide contextual comparison rather than a strictly controlled head-to-head ranking.

Our SEED LOSO accuracy of 57.95% is numerically higher than the listed SVM baseline and close to the pretrained-CNN result under three-class evaluation. However, differences in preprocessing, input representation, training strategy, and evaluation procedures should be considered when interpreting these comparisons. The source-free domain adaptation result uses a different adaptation strategy, the PDPL method relies on handcrafted EEG features, and the result reported on the DENS dataset was obtained using a different dataset. Therefore, Table 13 provides contextual rather than strictly controlled comparisons.

These results collectively confirm that subject-independent EEG classification at high accuracy remains an open problem regardless of architectural sophistication. The 40.57 percentage-point gap between our subject-dependent (98.52%) and subject-independent (57.95%) results on SEED quantifies the practical cost of cross-subject generalization and motivates future work on domain adaptation and transfer learning.

### 6.3. Subject-Dependent vs. Subject-Independent Gap

Table 14 summarizes the reported subject-dependent and subject-independent accuracies and quantifies the corresponding performance gap for each dataset.

**Figure 6:**
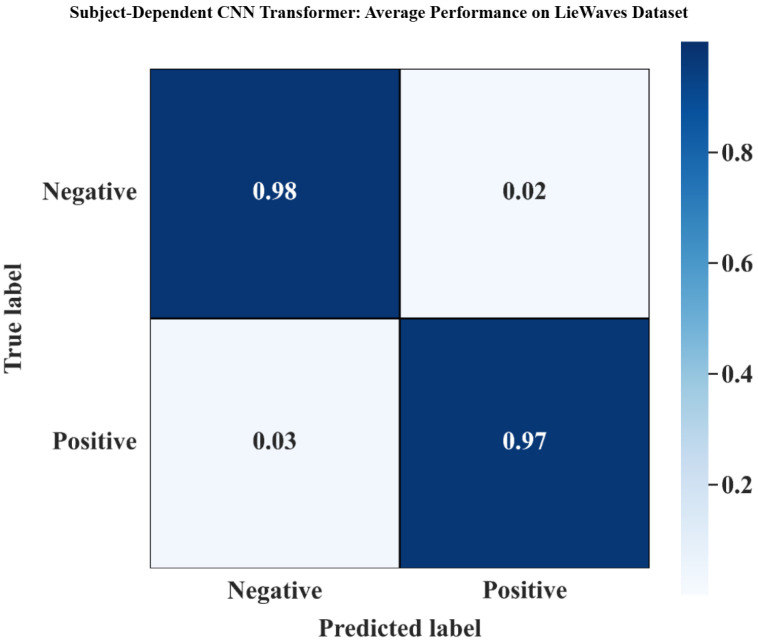
Absolute-count confusion matrix of the CNN–Transformer model on the LieWaves dataset under subject-dependent evaluation.

The subject-dependent LieWaves confusion matrix is shown in Figure 6.

The reported subject-dependent-to-subject-independent gaps were 38.29 percentage points for LieWaves and 40.57 percentage points for SEED. These findings are consistent with the substantial inter-subject variability documented in EEG research [1,2,15]. Although subject-dependent performance is high, the lower LOSO accuracy demonstrates that the learned representations do not transfer equally well to unseen subjects. This finding is consistent with the substantial inter-subject variability documented in EEG research [1,2,16]. Although subject-dependent performance is high, the lower LOSO accuracy demonstrates that the learned representations do not transfer equally well to unseen subjects.

## 7. Discussion

### 7.1. Architecture-Density Co-Design

The results demonstrate the feasibility of selecting different architectures for EEG datasets with substantially different channel-density regimes. The CNN–Transformer is effective on LieWaves precisely because the 5-channel input provides limited spatial redundancy, making temporal modeling the primary discriminative challenge. The six-layer Transformer encoder captures long-range dependencies across the 1 s epoch that a purely convolutional model would miss, while the lightweight parameter budget (≈850 k) prevents overfitting on a per-subject training set that is small by deep learning standards.

Conversely, the Inception CNN is effective on SEED because the 62-channel input already encodes rich spatial structure; the architectural bottleneck shifts from temporal range to temporal scale. The parallel branches with kernel sizes {3,5,7} capture local temporal patterns at multiple short time scales without requiring additional depth, and the lateral widening strategy keeps the parameter count at 0.49 M despite the higher input dimensionality. Because both architectures were not evaluated on both datasets under the same protocol, the present study does not establish that channel density alone determines the optimal architecture. A controlled cross-architecture comparison remains future work.

### 7.2. Augmentation: Mean Accuracy vs. Robustness

The effect of augmentation differed across datasets and evaluation settings. As shown in Figure 5, subject-dependent accuracy on SEED increased by only 0.08 percentage points, and neither the paired accuracy difference nor the difference in inter-subject dispersion was statistically significant.

In contrast, Figure 3 shows that augmentation increased subject-dependent LieWaves accuracy by 4.23 percentage points and significantly reduced inter-subject dispersion. However, this improvement did not extend to the subject-independent setting. As summarized in Table 14, LOSO accuracy changed from 58.85±9.10% without augmentation to 57.83±8.96% with augmentation, and the paired difference was not statistically significant.

**Figure 7:**
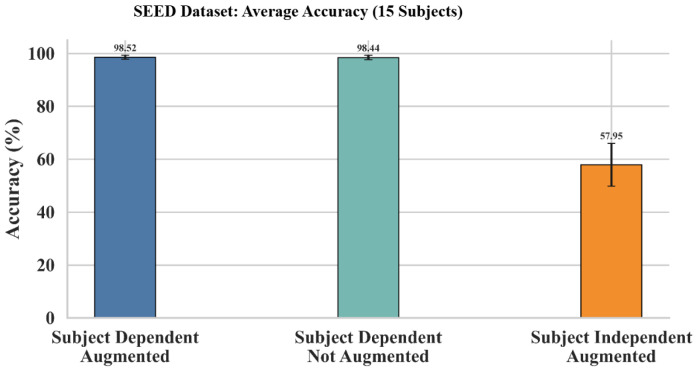
Comparison of model accuracy on the SEED dataset across different conditions.

The SEED accuracies across the evaluated conditions are compared in Figure 7.

### 7.3. The Subject-Independent Gap and Its Implications

The large gap between subject-dependent and subject-independent performance reflects the well-documented difficulty of learning EEG representations that generalize across individuals. EEG signals are shaped by individual differences in cortical geometry, skull thickness, electrode impedance, and cognitive strategy, all of which produce systematic distributional shifts between subjects that a model trained on one group cannot fully compensate for [16]. The fact that this gap is consistent across two architectures, two tasks, and two datasets of different channel densities reinforces that it is a property of the data rather than a correctable modeling artifact.

**Figure 8:**
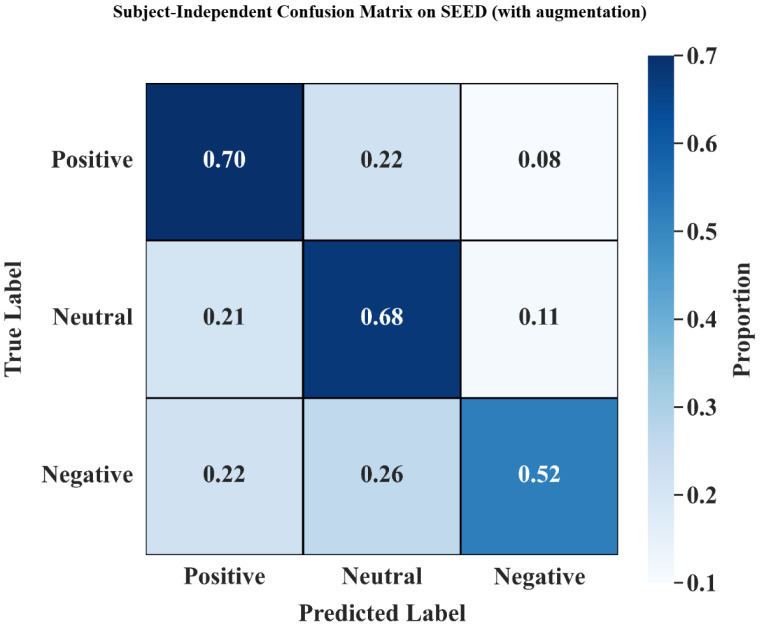
Absolute-count confusion matrix of the Inception CNN on the SEED dataset under LOSO evaluation.

This has a direct implication for deployment. Subject-dependent models are appropriate when a brief per-user calibration session is feasible, which may be a reasonable assumption in clinical BCI, rehabilitation robotics, and forensic assessment applications where setup time is acceptable. Subject-independent models are necessary when calibration is impractical, such as in passive affective monitoring or large-scale consumer applications, but the current state of the art including our own results suggests that acceptable accuracy in those settings requires methods beyond standard end-to-end deep learning. The class-wise performance of the Inception CNN under SEED LOSO evaluation is shown in Figure 8.

### 7.4. Pathways Toward Improved Subject-Independent Performance

Two methodological directions show the most promise for closing the subject-independent gap based on the recent literature.

Multi-source transfer learning [19]. Rather than training a single model on all source subjects, this approach selects the subset of source subjects whose EEG distribution most closely matches the target subject and fine-tunes on a small labeled calibration set from the target. Prior work reports improvements of up to 12.7% over non-transfer baselines on SEED, suggesting that compatibility-aware source selection is more effective than pooling all available data indiscriminately.

Domain-adversarial adaptation [18]. By adding a gradient-reversal layer that penalizes subject-discriminative features during training, domain-adversarial models learn representations that are predictive of the label but invariant to subject identity. When combined with pseudo-label refinement on unlabeled target data, this approach has demonstrated strong results on SEED and DEAP benchmarks. Integrating domain-adversarial training into the Inception CNN backbone proposed here is a natural extension that we leave for future work.

Both directions require either labeled calibration data or unlabeled target recordings at test time, which introduces its own practical constraints. A longer-term goal is zero-shot cross-subject generalization, which would require either very large and diverse EEG pretraining corpora or explicit neurophysiological priors encoded into the model architecture.

### 7.5. Limitations

Several limitations constrain the generalizability of the present findings and should be addressed in future work.

Dataset scale. Both SEED (15 subjects) and LieWaves (27 subjects) are small by the standards of modern deep learning benchmarks. Subject-dependent results on datasets of this size are susceptible to high variance across random seeds and initialization, even after early stopping and checkpoint selection. The low standard deviation observed on SEED (0.79) is encouraging, but replication on larger cohorts is necessary before strong generalization claims can be made.

Session-to-session non-stationarity. EEG is non-stationary: electrode impedance, fatigue, attention, and environmental noise vary across recording sessions even for the same subject. All experiments in this work are conducted within a single session per subject, meaning session-to-session drift is not characterized. Real-world deployment would require either frequent recalibration or online adaptation to maintain the accuracy levels reported here.

Computational constraints of the Transformer. The self-attention mechanism scales quadratically with sequence length, which limits the CNN–Transformer’s applicability to longer EEG epochs without architectural modification. For wearable or edge deployment, the Transformer encoder may require distillation or pruning to meet latency and memory constraints.

Scope of augmentation evaluation. The proposed augmentation strategy was evaluated on two datasets representing binary and three-class classification. Its effectiveness on tasks with more classes, longer epochs, or different noise characteristics, such as motor imagery EEG, has not been established and warrants independent validation.

### 7.6. Broader Applicability

Although validated on SEED and LieWaves, the proposed pipeline is dataset-agnostic in its design. The Inception CNN can be applied to any high-density EEG benchmark by adjusting the input channel dimension; the CNN–Transformer is appropriate for any low-channel setting by modifying the CNN encoder’s input size. The augmentation strategy requires only that a Butterworth filter can be applied to the raw signal, which holds for any EEG recording with a sampling rate above 90 Hz. Direct application to DEAP and AMIGOS, both of which provide multichannel emotion-elicitation EEG, is straightforward and represents a natural next step for validating the generality of these findings.

## 8. Conclusions

This study presented a sensor-density-aware EEG classification framework consisting of a CNN–Transformer for low-channel LieWaves recordings and an Inception CNN for high-channel SEED recordings. The two architectures were combined with a signal-level augmentation strategy that expands the training set by transferring high-frequency components above 45 Hz from reference EEG recordings to cleaned source recordings while largely preserving the lower-frequency signal structure.

Under subject-dependent evaluation, augmentation increased LieWaves accuracy from 92.91±4.34% to 97.14±1.36%, corresponding to an absolute improvement of 4.23 percentage points. The paired improvement was statistically significant, and augmentation also significantly reduced inter-subject dispersion. In contrast, SEED accuracy changed only from 98.44±0.83% without augmentation to 98.52±0.79% with augmentation. Neither the paired accuracy difference nor the difference in inter-subject dispersion was statistically significant, indicating that augmentation provided limited additional benefit when baseline performance was already near ceiling.

Under subject-independent evaluation, performance remained substantially lower. On LieWaves, LOSO accuracy changed from 58.85±9.10% without augmentation to 57.83±8.96% with augmentation, and the difference was not statistically significant. On SEED, the Inception CNN achieved 57.95±8.38% under LOSO evaluation. These results demonstrate that the proposed models provide strong subject-dependent performance but remain limited in their ability to generalize to unseen subjects.

Overall, the proposed framework provides a practical comparison of sensor-density-aware architecture selection, signal-level augmentation, and subject-dependent versus subject-independent EEG classification. The results show that augmentation is particularly effective for subject-dependent LieWaves classification but provides limited benefit on SEED and does not improve cross-subject LieWaves performance. Future work should investigate domain adaptation, transfer learning, subject-invariant feature learning, and larger multi-subject EEG datasets to improve generalization to unseen users.

## Figures and Tables

**Table 1:** Motivation, novelty, aim, and objectives of the proposed study.

ID	Objective	Target/Expected Outcome
O1	Design a low-channel EEG classification model	Develop a lightweight CNN–Transformer architecture for the 5-channel LieWaves deception detection task.
O2	Design a high-channel EEG classification model	Develop an Inception-based CNN for the 62-channel SEED emotion recognition task.
O3	Assess augmentation under limited EEG data	Quantify the effect of signal-level augmentation on mean accuracy and inter-subject variability in both subject-dependent and subject-independent settings.
O4	Evaluate subject-dependent performance	Report accuracy, precision, recall, F1-score, standard deviation, and absolute-count confusion matrices for within-subject evaluation.
O5	Evaluate subject-independent generalization	Use leave-one-subject-out (LOSO) evaluation to quantify the cross-subject performance gap.
O6	Improve reproducibility and interpretability	Report dataset splits, augmentation settings, hyperparameter choices, architectural complexity, statistical tests, and practical limitations.

**Table 2:** Limitations in existing EEG classification studies and corresponding responses in this work.

Research Direction	Observed Limitation	Response in This Work
Handcrafted ERP/spectral features	Many methods depend on manually engineered ERP, DWT, or band-power features, which may be task-specific and difficult to reproduce across datasets.	The proposed models learn directly from segmented EEG epochs, reducing dependence on handcrafted feature pipelines.
CNN/LSTM EEG classifiers	Convolutional and recurrent models capture local or sequential patterns but may not explicitly model long-range temporal dependencies.	The low-channel LieWaves model combines CNN feature extraction with Transformer self-attention.
GAN-based augmentation	Synthetic samples may drift from the physiological EEG manifold or introduce label-inconsistent artifacts.	The proposed augmentation uses real EEG trials and injects only high-frequency noise components into cleaned signals.
Single-architecture studies	Prior work often applies one model type regardless of channel density or acquisition regime.	We use a CNN–Transformer for low-channel EEG and an Inception CNN for high-channel EEG.
Evaluation protocol reporting	Some studies emphasize subject-dependent performance without adequately reporting cross-subject generalization.	Both subject-dependent and LOSO subject-independent protocols are reported.
Reproducibility details	Layer-wise characteristics, dataset splits, augmentation counts, and hyperparameter tuning are often omitted.	The revised manuscript provides explicit architecture, split, augmentation, and hyperparameter tables.

**Table 3:** Layer-wise specification of the CNN–Transformer architecture for LieWaves. Parameter counts correspond to the described six-layer model with input shape (B,5,128).

Stage	Functional Role	Input	Output	Trainable Params
Conv1d + BN + ReLU + Pool	Coarse temporal feature extraction and downsampling	(B,5,128)	(B,32,·)	1216
Conv1d + BN + ReLU + Pool	Mid-level temporal representation learning	(B,32,·)	(B,64,·)	10,432
Conv1d + BN + ReLU + Pool	Local discriminative feature refinement	(B,64,·)	(B,128,·)	24,960
Adaptive AvgPool1d	Fixed-length token sequence formation	(B,128,·)	(B,128,60)	0
Linear projection	Token embedding projection	(B,60,128)	(B,60,128)	16,512
Transformer Encoder ×6	Long-range temporal dependency modeling using 8-head self-attention	(B,60,128)	(B,60,128)	794,880
Mean pooling	Sequence aggregation	(B,60,128)	(B,128)	0
LayerNorm + MLP Classifier	Binary truth/lie prediction	(B,128)	(B,1)	8577
**Total**	–	–	–	**856,577 (approximately 0.86 M)**

**Table 4:** Stage-wise shape transformations in the Inception model for SEED (T=400).

Stage	Input Shape	Output Shape
Input	(B,62,400)	(B,62,400)
Inception Block 1	(B,62,400)	(B,96,400)
MaxPool1d	(B,96,400)	(B,96,200)
Inception Block 2	(B,96,200)	(B,192,200)
MaxPool1d	(B,192,200)	(B,192,100)
Inception Block 3	(B,192,100)	(B,384,100)
GlobalAvgPool1d	(B,384,100)	(B,384)
Fully Connected	(B,384)	(B,3)

**Table 5:** Layer-wise characteristics of the SEED Inception CNN. Parameter counts are computed from the implemented architecture.

Layer/Stage	Functional Role	Input	Output	Trainable Params
Inception Block 1	Multi-scale temporal feature extraction using kernels 3, 5, and 7	(B,62,400)	(B,96,400)	30,048
MaxPool1d	Temporal downsampling	(B,96,400)	(B,96,200)	0
Inception Block 2	Mid-level multi-scale temporal abstraction	(B,96,200)	(B,192,200)	92,736
MaxPool1d	Temporal downsampling	(B,192,200)	(B,192,100)	0
Inception Block 3	High-level temporal-spatial representation learning	(B,192,100)	(B,384,100)	369,792
GlobalAvgPool1d	Temporal aggregation	(B,384,100)	(B,384)	0
Fully Connected	Three-class emotion classification	(B,384)	(B,3)	1155
**Total **	–	–	–	**493,731**

**Table 6:** Computational profile of the Inception CNN for a single SEED input window. Latency was measured with batch size 1.

Metric	Value
Input shape	(1,62,400)
Trainable parameters	493,731
Model size	1.889 MB
MACs per input	67.70 million
Mean inference latency	0.680±0.121 ms
Peak GPU memory	11.13 MB

**Table 7:** Summary of datasets used in this study.

Characteristic	LieWaves	SEED
Task	ERP/P300-based deception detection	EEG-based emotion recognition
Classes	Truth and lie	Negative, neutral, and positive
Subjects	27	15
Channels	5 channels (AF3, T7, Pz, T8, AF4)	62 channels
Sampling rate used	128 Hz	Provider-released recordings downsampled to 200 Hz
Input epoch/window	(5,128), 1-s epochs	(62,400), 2-s windows
Segmentation	25% overlap, stride 96 samples	50% overlap, stride 200 samples
Primary model	CNN–Transformer	Inception CNN
Evaluation protocols	Subject-dependent and LOSO	Subject-dependent and LOSO
Ethical consideration	De-identified dataset collected in the original LieWaves study	Public de-identified SEED dataset released for academic research

**Table 8:** Dataset size before and after augmentation. Augmentation is applied only to the training split; validation and test/held-out subject data remain unchanged. Symbols $N_{\mathrm{train}}$, $N_{\mathrm{val}}$, and $N_{\mathrm{test}}$ denote the number of windows/epochs generated by the corresponding split in each evaluation fold.

Dataset	Split	Before Augmentation	After Augmentation
LieWaves	Training	$N_{\mathrm{train}}$	$4N_{\mathrm{train}}$
LieWaves	Validation	$N_{\mathrm{val}}$	$N_{\mathrm{val}}$
LieWaves	Test/LOSO held-out subject	$N_{\mathrm{test}}$	$N_{\mathrm{test}}$
SEED	Training	$N_{\mathrm{train}}$	$4N_{\mathrm{train}}$
SEED	Validation	$N_{\mathrm{val}}$	$N_{\mathrm{val}}$
SEED	Test/LOSO held-out subject	$N_{\mathrm{test}}$	$N_{\mathrm{test}}$

**Table 9:** Hyperparameter search space and final selected values.

Hyperparameter	Search Values	CNN–Transformer	Inception CNN
Optimizer	Adam	Adam	Adam
Learning rate	$\left\{10^{-4},5\times 10^{-4},10^{-3},5\times 10^{-3}\right\}$	$10^{-4}$	$5\times 10^{-4}$
Batch size	{16,32,64}	32	32
Weight decay	$\left\{0,10^{-3},10^{-2}\right\}$	$10^{-2}$	$10^{-2}$
Dropout	{0.1,0.2,0.3,0.5}	0.2 encoder, 0.3 head	empirical setting from final run
Transformer heads	{4,8,16}	8	N/A
Transformer layers	{2,4,6,8}	6	N/A
Augmented copies	{1,2,3,4}	3	3
Loss function	Task dependent	Binary cross-entropy	Categorical cross-entropy
Early stopping patience	{5,10,20}	10	10

**Table 10:** Training, validation, and testing protocols.

Evaluation Setting	Training Split	Validation/Test Split	Leakage Prevention
Subject-dependent	Per-subject trial-level training split with stratified class proportions	Held-out trials from the same subject; no overlapping trials	Split is performed before augmentation; only training data are augmented.
Subject-independent LOSO	All subjects except one held-out test subject	One completely unseen subject per fold	No subject appears in both training and test data.
SEED internal validation	Training windows from selected trials/subjects	Validation windows are kept unaugmented	Normalization statistics are fitted on training data only.
LieWaves internal validation	Training epochs from selected trials/subjects	Validation epochs are kept unaugmented	Trial-level separation prevents duplicated epoch leakage.

**Table 11:** Subject-dependent performance on SEED. Mean accuracy, standard deviation, and macro F1-score are reported. Dashes indicate metrics not reported in the original studies; therefore, comparisons involving missing variance or F1-score values should be interpreted cautiously.

Model	Acc. (%)	Std	F1
SPDNet [24]	90.63	—	—
3D-CNN + ELM [22]	90.85	14.72	0.89
CNN-KAN [25]	97.45	—	0.97
Deep Capsule Network [23]	98.21	—	—
**Inception CNN (ours) **	**98.52**	**0.79**	**0.98**

**Table 12:** Subject-dependent performance on LieWaves.

Model	Acc. (%)	F1
CNN–LSTM + DWT [2]	98.76	—
**CNN–Transformer (ours)**	**97.14**	**0.97**

**Table 13:** Contextual comparison of subject-independent EEG classification results. Differences in dataset, class formulation, input representation, and evaluation protocol should be considered when interpreting the reported accuracies.

Model	Acc. (%)	Note
SVM baseline [26]	56.00	3-class, SEED
Pretrained CNN [27]	58.10	3-class, SEED
**Inception CNN (ours)**	**57.95**	3-class SEED, LOSO, augmentation
Source-free DA [28]	65.84	2-class, SEED
PDPL [29]	69.89	Handcrafted features
CNN (DENS dataset) [30]	73.04	Different dataset

**Table 14:** Summary of subject-dependent and subject-independent accuracy (%) across the two datasets.

Dataset	SD, No Aug.	SD, Aug.	SI (LOSO)	Gap
LieWaves	92.91	97.14	58.85	38.29
SEED	98.44	98.52	57.95	40.57

SD = subject-dependent; SI = subject-independent; Gap = SD with augmentation − reported SI accuracy.

## Data Availability

The EEG datasets used for training and evaluation are publicly available as follows: The SEED (SJTU Emotion EEG Dataset) is available from the BCMI Laboratory at SJTU (available at https://bcmi.sjtu.edu.cn/home/seed/, accessed on 28 March 2025). The LieWaves dataset is available upon request or via its official repository (available at https://data.mendeley.com/datasets/5gzxb2bzs2/2, accessed on 28 March 2025). These datasets are subject to the data usage and license policies of their respective providers.

## Abbreviations

The following abbreviations are used in this manuscript:

BCI	Brain–Computer Interface
CNN	Convolutional Neural Network
DA	Domain Adaptation
DEAP	Database for Emotion Analysis using Physiological Signals
DWT	Discrete Wavelet Transform
EEG	Electroencephalography
ELM	Extreme Learning Machine
ERP	Event-Related Potential
F1	F1-Score (Harmonic Mean of Precision and Recall)
GAN	Generative Adversarial Network
GLM	General Linear Model
LOSO	Leave-One-Subject-Out
LSTM	Long Short-Term Memory
MLP	Multi-Layer Perceptron
PDPL	Projection Dictionary Pair Learning
ReLU	Rectified Linear Unit
RNN	Recurrent Neural Network
SD	Subject-Dependent
SDR	Standard Deviation Ratio
SEED	SJTU Emotion EEG Dataset
SI	Subject-Independent
SPD	Symmetric Positive Definite
SVM	Support Vector Machine
